# The Advance of Magnetic Resonance Elastography in Tumor Diagnosis

**DOI:** 10.3389/fonc.2021.722703

**Published:** 2021-08-31

**Authors:** Jin-Ying Yang, Ben-Sheng Qiu

**Affiliations:** ^1^Laboratory Center for Information Science, University of Science and Technology of China, Hefei, China; ^2^Hefei National Lab for Physical Sciences at the Microscale and the Centers for Biomedical Engneering, University of Science and Technology of China, Hefei, China

**Keywords:** noninvasive, hardness, stiffness, tumor diagnosis, MRE

## Abstract

The change in tissue stiffness caused by pathological changes in the tissue’s structure could be detected earlier, prior to the manifestation of their clinical features. Magnetic resonance elastography (MRE) is a noninvasive imaging technique that uses low-frequency vibrations to quantitatively measure the elasticity or stiffness of tissues. In tumor tissue, stiffness is directly related to tumor development, invasion, metastasis, and chemoradiotherapy resistance. It also dictates the choice of surgical method. At present, MRE is widely used in assessing different human organs, such as the liver, brain, breast, prostate, uterus, gallbladder, and colon stiffness. In the field of oncology, MRE’s value lies in tumor diagnosis (especially early diagnosis), selection of treatment method, and prognosis evaluation. This article summarizes the principle of MRE and its research and application progress in tumor diagnosis and treatment.

## Introduction

**Figure 1:**
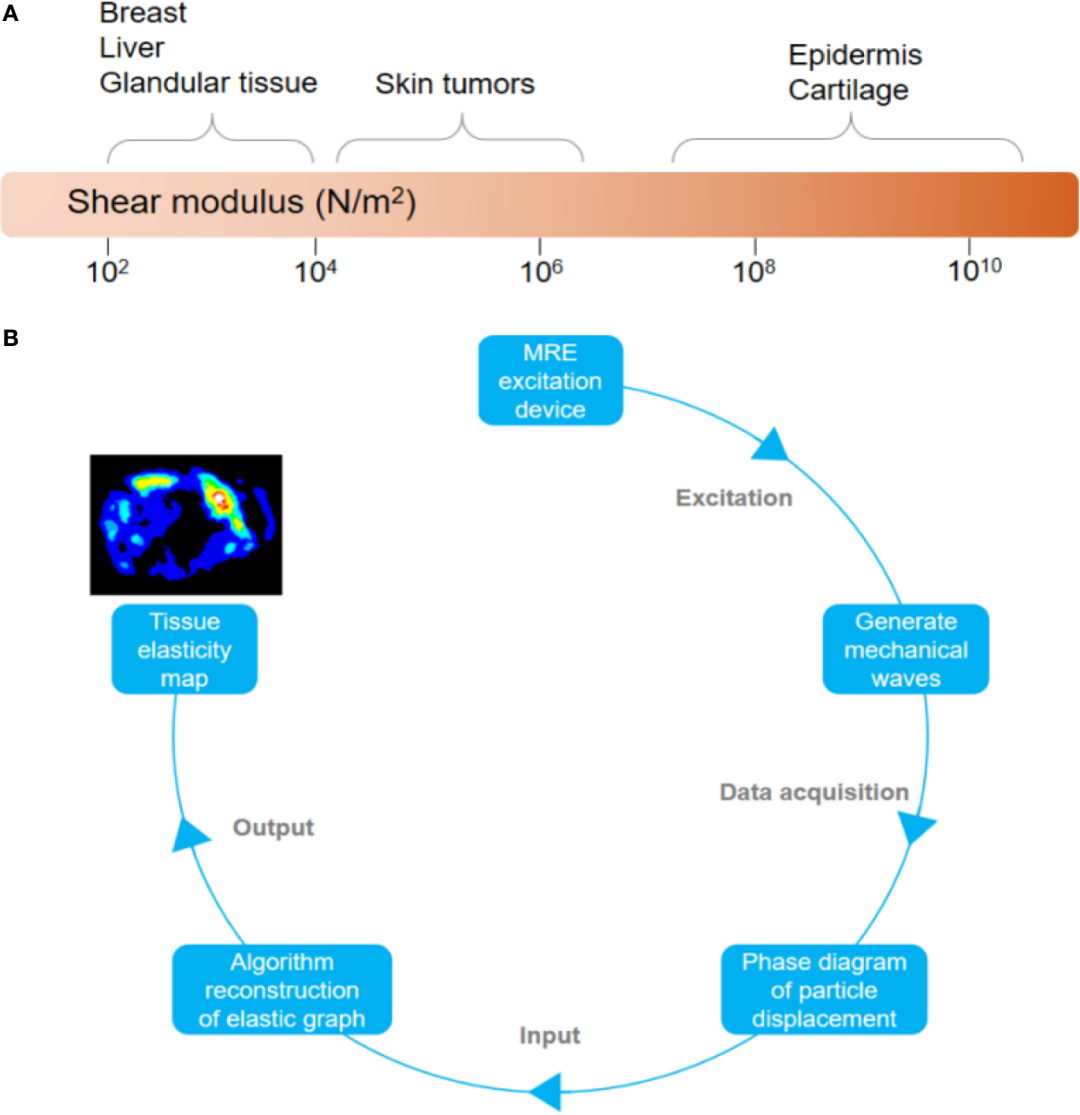
**(A)** Elastic value of some human tissues. **(B)** Flowchart of magnetic resonance elastography.

Stiffness is an important mechanical parameter and one of the physical properties of human tissues, closely related to biological characteristics (1, 2). Different tissues or organs have different degrees of stiffness (see **Figure 1A**). It was found that the elasticity of tumor cells (0.05-3.0 kPa) was lower than that of normal human cells (0.75-90 kPa), while the stiffness of tumor tissue is higher than that of normal tissue (3, 4). This is due to the rapid proliferation of tumor cells and increased cell density after normal tissues have become cancerous; at the same time, a large amount of microvascular reconstruction and reduction of normal gland structure has resulted in tumor tissues becoming harder and tougher than the surrounding normal tissues (5). The infiltration of tumor tissue into the surrounding tissue also leads to increased collagen deposition (6, 7). In addition, microvascular pressure, abnormal blood flow, lymphatic and vascular leakage, and increased osmotic pressure in the interstitial space are also factors that increase the stiffness of tumor tissues. In sum, the abnormal structure and composition increased the stiffness of the tumor tissue. Therefore, studying the stiffness of tumor tissue leads to better understanding of its features and behavior, especially in terms of local invasion, distant metastasis, and chemoradiotherapy resistance. More importantly, it will influence the choice of surgical method to be used as part of the treatment (8–10).

Elastography, also known as “palpation by imaging,” was proposed by Ophir et al. in 1991 (11). It was first used in ultrasound, but its accuracy in assessing tissue elasticity was poor due to factors such as increased subjectivity and lack of uniform operating specifications. MRE is a noninvasive imaging technique that quantifies the elastic characteristics of tissues. It has been used in clinical research and application that involve different kinds of tumors. It is considered an ideal diagnostic tool because it is safe to use, noninvasive, highly repeatable, produces reliable results, less operator-dependent, and provides a clear, highly detailed images of tumors (12).

## Principle of MRE

MRE is based on magnetic resonance technology. First, it applies continuous and dynamic mechanical shear waves to the target tissue through an exciter. Then, the motion-sensitive gradient of magnetic resonance (MR) is used to obtain the phase distribution information of the mass points in the tissue. By doing this, the phase and wave patterns of the dynamic shear wave propagation are obtained. Finally, both the full amplitude and full quantitative color-coded elastic graph are generated by the inversion algorithm of the waveform map through the elastic imaging software (see **Figure 1B**) (8, 13). The MRE imaging process can be summarized as follows: 1) Shear waves are generated in the tissue; 2) MR images reflecting the propagation of the shear waves are created; 3) After processing the shear wave image, the elastogram quantifying the stiffness of the tissue is obtained (9). By selecting any region of interest (ROI) in the graph, the elasticity value (in kiloPascal or kPa) in the region can be obtained to quantitatively evaluate the elasticity attribute. MRE can combine different magnetic resonance imaging (MRI) sequences to acquire signals, such as gradient echo (GRE), spin-echo (SE), spin-echo echo-planar imaging (SE-EPI), and balanced steady-state free precession (bSSFP) (13–16). At present, the most commonly used clinically is the traditional 60 Hz 2D gradient echo (GRE) sequence. Compared with the GRE sequence, SE-EPI has the advantage of short scanning time, high image quality and success rate of tumor detection. This makes it highly favorable to researchers (17, 18). MRE that combines SE-EPI sequence and 3D increases spatial coverage and reduces the errors caused by oblique wave propagation and edge artifacts, so the measured values obtained are more stable and accurate (19, 20). In addition, the SE-EPI sequence is less sensitive to liver iron overload and requires less patient cooperation, both of which make obtaining data faster (21). 3D-MRE’s current scanning time is long, which makes it less ideal to use (19). However, at the rate that technological advances are happening right now, it would not be long before 3D-MRE becomes the main application of MRE.

## Application of MRE in Tumor Diagnosis and Treatment

Changes in the elasticity and stiffness of human tissues reflect the pathological development process to a certain extent (22). For example, 80–90% of patients with liver cirrhosis will develop liver cancer, with tumor stiffness directly related to tumor grade (23–25). In addition, high tumor stiffness hinders the effective delivery of anti-cancer drugs in the body (26–28). Accurately identifying and reducing elastic stiffness between tissues contribute to the efficient delivery of drugs in patients undergoing treatment (29, 30). MRE technology provides a new strategy for the precise diagnosis and treatment of tumors.

### Liver Cancer

Currently, MRE is extensively applied in the diagnosis of liver diseases. It is used in accurately assessing normal liver parenchyma, liver fibrosis, focal nodular hyperplasia, and liver cancer (22). MRE effectively identifies the stages of liver fibrosis and detects early liver cirrhosis (see **Table 1**). The sensitivity of MRE in distinguishing between severe and mild liver fibrosis is 98%. The increase in liver stiffness in patients with liver cirrhosis is an important risk factor for developing hepatocellular carcinoma (HCC) (24).

**Table 1:** Liver shear stiffness (kPa) under different physiological or pathological conditions.

Different physiological or pathological conditions of liver		shear stiffness (kPa)					Reference
		F0	F1	F2	F3	F4	
Non-alcoholic fatty liver disease		2.36	2.76	3.36	4.56	5.68	(31–36)
Hepatitis C virus		2.10	2.42	3.16	4.22	6.21	(37, 38)
Hepatitis B virus		2.52	2.88	3.46	4.35	6.54	(37, 39)
Autoimmune hepatitis	untreated	3.10	2.94	3.20	4.10	6.50	(40)
	treated	2.61	2.74	2.63	3.99	5.90	
Primary sclerosing cholangitis			3.49	3.68	3.84	4.11	(41)
Alcoholic liver disease			2.20	2.57	3.31	4.00	(42)
Cholestatic		3.53	2.76	4.00	3.91	6.38	(37, 43)
Portal Hypertension			HVPG <5mmHg	HVPG ≥5mmHg	HVPG <10mmHg	HVPG ≥10mmHg	(44)
			2.31	5.14	3.88	5.86	

F0-F4 fibrosis stage; HVPG hepatic venous pressure gradient.

**Figure 2:**
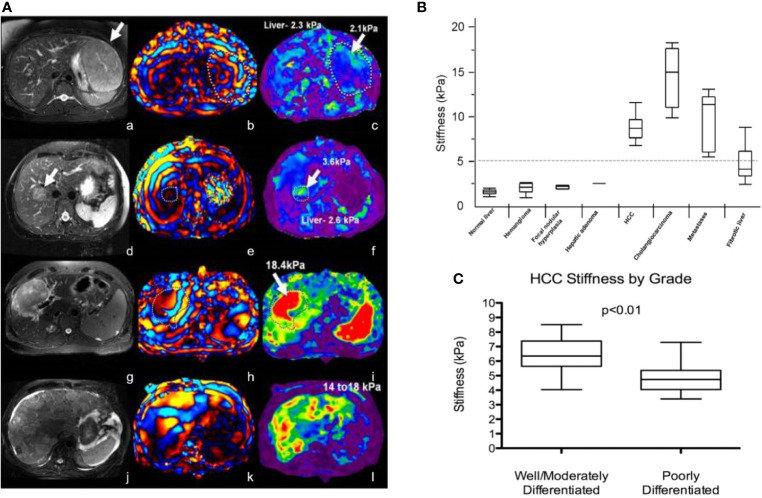
**(A)** MRE of liver tumors. The stiffness value of benign tumors (focal nodular hyperplasia, a-c; liver adenoma, d-f) is equivalent to that of normal liver parenchyma (generally less than 5 kPa), while the stiffness of malignant tumors (hepatocellular carcinoma-cholangiocarcinoma, g-i; colorectal cancer metastasis, j-l) is increased Reproduced with permission from ref. (46), copyright 2013, John Wiley & Sons Inc. **(B)** Shear stiffness of different tissues. Reproduced with permission from ref. (45), copyright 2008, American Roentgen Ray Society. **(C)** Schematic diagram of HCC tumor grade corresponding to tumor stiffness (kPa). Reproduced with permission from ref. 23, copyright 2017, Elsevier.

Richard et al. evaluated 29 patients with 44 liver tumors using MRE with improved gradient echo sequence. The results showed that the average stiffness of malignant liver tumor (10.1 kPa) was higher than that of benign liver tumor (2.7 kPa), liver fibrosis (5.9 kPa), and normal liver parenchyma (2.3 kPa) (45). There was no significant difference in shear stiffness between benign liver tumors and normal liver parenchyma. They initially determined that the stiffness value of the liver was 5 kPa, which was the critical value that distinguishes malignant liver tumor from benign liver tumor or normal liver parenchyma (see **Figures 2A, B**).

Garteiser et al. found that the loss modulus of hepatocellular carcinomas was significantly higher than that of benign hepatocellular tumors (47). In addition, MRE can also be used to initially assess the level of hepatocellular carcinoma (HCC). For example, compared with poorly differentiated liver cancer (4.9 ± 1.2 kPa; N = 8), well-differentiated/moderately differentiated HCC (6.5 ± 1.2 kPa; N = 13) tumor stiffness increased significantly (see **Figure 2C**). However, there is no exact correlation between the stiffness of liver tumors and tumor size. MRE is a promising diagnostic technique for evaluating solid liver tumors.

Liver stiffness measured by MRE can be used to predict the early recurrence of liver tumors after treatment. The stiffness of liver tumors is an independent factor in the early recurrence of HCC (48). For every 1 kPa increase in tumor stiffness, the risk of tumor recurrence increased by 16.3%. The relapsed HCC has higher tumor stiffness (49). Liver stiffness measured by MRE can also be used as a prognostic indicator for HCC patients undergoing hepatectomy. Liver stiffness (≥ 4.02 kPa) was the only important factor for poor overall survival (OS) (50). Meanwhile, the value of liver stiffness is negatively correlated with the regenerating ability of the residual liver after hepatectomy (51). Therefore, liver stiffness measured by MRE can be used to predict liver regeneration in patients with liver cirrhosis and liver cancer. Moreover, MRE data of patients with colorectal liver metastases treated by transcatheter arterial chemoembolization (TACE) showed that the stiffness of the metastases was higher (P< 0.001) (52). MRE provides a reference value for the treatment of patients with liver metastasis.

### Breast Cancer

**Figure 3:**
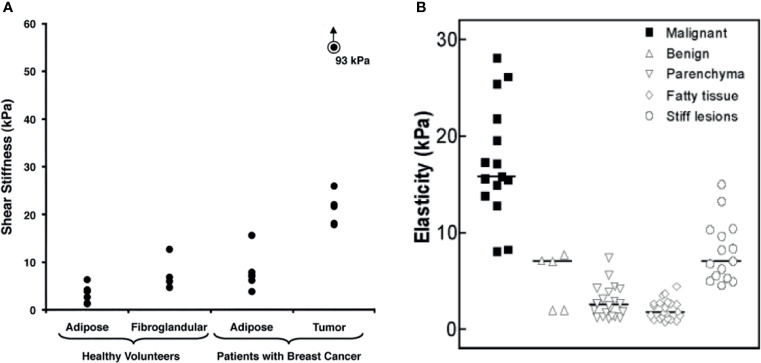
**(A)** Shear stiffness estimates of different types of breast tissue (mean value). Reproduced with permission from ref. (53), copyright 2002, American Roentgen Ray Society; **(B)** MRE–based elasticity values for malignant, benign, parenchyma, fatty, and stiff lesions (median). Reproduced with permission from ref. (54), copyright 2002, Georg Thieme Verlag KG.

MRE imaging of the breast requires low-frequency emission (generally, 40–100 Hz). Ehman et al. detected MRE with 100 Hz shear wave in healthy volunteers and breast cancer patients. The results showed that the average elastic values of normal adipose tissue, fibroglandular tissue, and tumor tissue were 3.3 kPa, 7.5 kPa, and 33 kPa, respectively (see **Figure 3A**) (53). Between these elastic values, the stiffness of breast cancer tissue is four times that of normal fibroglandular tissue. Meanwhile, Lorenzen et al. found that the median elasticity of breast adipose tissue, breast parenchyma, benign tumor tissue, and malignant tumor tissue were 1.7 kPa, 2.5 kPa, 7.0 kPa, and 15.9 kPa, respectively (see **Figure 3B**) (54). The elasticity value of breast cancer tissue was higher than that of normal tissue around the tumor, benign tumor, and normal breast tissue (55).

Balleyguier et al. studied 43 breast tumor patients with high scores in the breast imaging report and data system (BI-RADS), and found that the sensitivity, specificity, positive predictive value, and negative predictive value of MRE for breast cancer diagnosis were 79%, 90%, 96%, and 56%, respectively. MRE with an AUC (Area under the curve) value of 0.92 as compared with 0.84 for MRI alone (56). Sinkus et al. observed an increase in specificity from 40 to 60% at 100% sensitivity, and Siegmann et al. also improved the specificity from 75% to 90% while maintaining a high sensitivity of 90% (57, 58). In these studies, MRE improved the accuracy of MRI in diagnosing breast cancer. This is because the elasticity of benign and malignant tumors overlaps. MRI alone cannot provide an accurate diagnosis; it needs to be combined with viscoelastic parameters (elasticity, viscosity, etc.) in order to provide a more comprehensive evaluation. It is also necessary to reduce scanning time and improve spatial resolution in the future to promote the clinical application of MRE in the diagnosis of breast cancer (59).

### Brain Tumor

MRE possesses significant clinical value as a tool in the diagnosis and treatment of brain tumors. The nondestructive conduction of shear waves into the skull is the key to this (60). Wuerfel et al. first discovered the correlation between brain stiffness and pathological process sensitivity in multiple sclerosis (MS). The average shear modulus of the white matter and gray matter of a normal human brain, independent of age, are 14.8 and 5.22 kPa, respectively (61).

**Figure 4:**
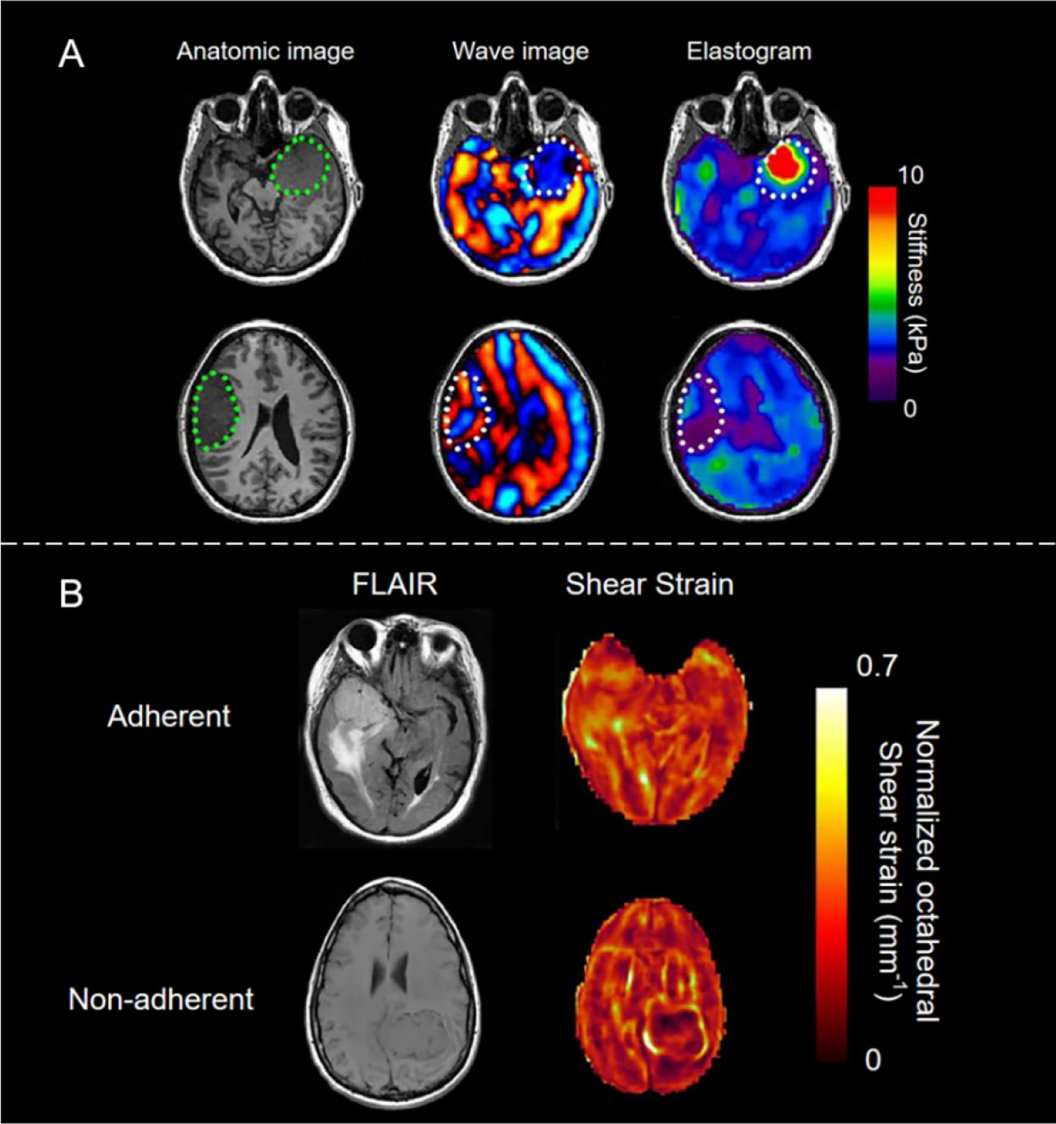
**(A)** Brain imaging of stiff meningioma subjects (top row) and soft meningioma subjects (bottom row). The area encircled by the green dots represents the location of the tumor. Normal brain parenchyma, dural meningioma, and soft meningioma have different mechanical properties, indicating the great potential of MRE in brain imaging. Reproduced with permission from ref. (62), copyright 2013, American Association of Neurological Surgeons. **(B)** The image of a sliding interface in the case of adherent (top) and non-adherent (bottom) meningiomas. In non-adherent cases, the tumor was surrounded by bright rings. Left: T2-weighted fluid-attenuated inversion recovery (FLAIR) images; Right: shear strain maps. Reproduced with permission from ref. (63).

One of the most important factors determining the difficulty of brain tumor resection is the consistency of the tumors. Several studies have shown that the shear stiffness of meningiomas and pituitary adenomas measured by MRE is closely related to the subjective assessment of tumor consistency by surgeons during surgery (62) (see **Figure 4A**). The sensitivity, specificity, positive predictive value, and negative predictive value of MRE for judging meningioma heterogeneity are 75%, 100%, 100%, and 87%, respectively; while the sensitivity, specificity, positive predictive value, and negative predictive value of MRE for judging tumor stiffness are 60%, 100%, 100%, and 56%, respectively (7). Another important factor affecting brain tumor resection is tumor adhesion. The slip interface imaging (SII) technology that was developed based on MRE is a noninvasive method of assessing the degree of adhesion of meningioma to adjacent brain tissues (63–65). SII’s ability to strongly predict tumor compliance indicates that it is a promising technique for surgical planning to help predict the duration and risk of surgery (see **Figure 4B**) (63).

### Prostate Cancer

Shear wave excitation devices for prostate MRE have been reported, such as transurethral excitation device, transrectal excitation device, and transperineal excitation device. These devices can observe uniform shear waves in animal and human experiments and have high rate of repeatability (66–69). The results of prostate MRE through perineal stimulation showed that the average elastic value of prostate cancer tissues was different from that of normal tissues, and their diagnostic sensitivity and specificity were 63% and 68%, respectively (5). Initial data showed that the average elasticity values of prostate cancer tissue, prostatitis tissue, and normal prostate tissue were 6.55 kPa, 1.99 kPa, and 2.26 kPa, respectively (70). Salcudean et al. developed a novel active shielding electromagnetic sensor and adopted a fast pulse sequence, which shortened the MRE data acquisition time to 8–10 minutes, making it more acceptable to patients (71).

Recently, Klatt et al. found that MRE can overcome some limitations of MRI in evaluating prostate cancer, such as interobserver variability and low specificity (72). In a 9.4T preclinical scanner, 14 patients with prostate cancer were examined using MRE at 500 Hz immediately after prostatectomy. MRE data showed that the average stiffness of tumor tissue and healthy tissue were 10.84 ± 4.65 and 5.44 ± 4.40 kPa (p ≤ 0.001), respectively, indicating that MRE is a highly promising imaging technique for diagnosing prostate cancer. The Youden index showed that the sensitivity and specificity of diagnosis were 69% and 79%, respectively. In addition, Wang et al. found that the detection of MRE-based prostate cancer (PCa) stiffness may help noninvasively predict the degree of lymph node metastasis prior to surgery, with sensitivity and specificity as high as 100% and 86.5%, respectively (73).

### Pancreatic Cancer

Among the main problems encountered regarding the early diagnosis of pancreatic cancer are early clinical symptoms that are not obvious and the unsuitableness of biopsy or laparotomy. Pancreatic cancer tumor cells infiltrate and grow into surrounding tissues, so they produce a large amount of collagen, which leads to increased stiffness. Generally, the stiffness of pancreatic cancer tissue is 6.06 ± 0.49 kPa, which is higher than that of normal pancreatic tissue (2.47 ± 0.11 kPa, P<0.0001) (74). Guo et al. found that the sensitivity, accuracy, and specificity of MRE in evaluating pancreatic cancer and pancreatitis were higher than 0.9, showing better diagnostic performance than carbohydrate antigen 19-9 (CA199) (75). Low frequency 40 Hz MRE combined with MRI can improve the specificity of diagnosis of pancreatic cancer (96.9% versus 62.1%, P = 0.002) (76). Hence, MRE has important research and clinical application value for the diagnosis and treatment of pancreatic cancer.

MRE has also been used in the diagnosis of myoma, colorectal cancer, and thyroid tumors (77–81). Overall research shows that MRE can accurately distinguish between benign and malignant tumors, which helps improve the specificity and sensitivity of tumor diagnosis.

## Conclusion and Prospect

MRE is a noninvasive technology that can improve the diagnosis and treatment of malignant tumors. It has gradually become a new research method in the field of oncology. MRE has the advantage of being highly accurate, producing clearer results, being highly repeatable, and having a high success rate. It can better assess the characteristics of malignant tumors, so that the best treatment and surgical methods are identified and applied. However, MRE technology still has many questions to be answered. Biological tissues filter the spectrum out of control, how does this frequency filter affect the measurement result? What is the role and relationship between stiffness, elasticity, and viscosity? The urgent challenge is to standardize the technology and to standardize the MRE examination and the “units” used. So far, elastography still without strict measurement conditions, MRE provides stiffness in kPa by calculating the shear modulus, while transient elastography provides Young’s modulus, which is nearly three times the shear modulus. Not to mention the limitations of the clinical application of tumor MRE: 1) vulnerability to respiratory factors and artifacts when diagnosing liver cancer and breast cancer; 2) limited ability to track calcified lesions; 3) low spatial resolution; 4) high iron content affecting data acquisition of liver MRE; 5) estimation error caused by different measurement parameters, such as field strength, scanning sequence, and shear wave frequency; 6) human error when processing and analyzing the image. Given these current limitations, it is necessary to further improve the MRE’s *in vitro* excitation device, optimize the acquisition parameters and stimulation frequency, and combine multiple parameters for comprehensive diagnosis. When these technical issues are addressed, with the application of artificial intelligence and machine learning in medical image processing, the MRE’s wider clinical application, especially in the field of oncology, will be fully realized.

## Author Contributions

J-YY: project idea and writing of the manuscript. J-YY and B-SQ: project idea, project planning, and review of the manuscript. All authors contributed to the article and approved the submitted version.

## Conflict of Interest

The authors declare that the research was conducted in the absence of any commercial or financial relationships that could be construed as a potential conflict of interest.

## Publisher’s Note

All claims expressed in this article are solely those of the authors and do not necessarily represent those of their affiliated organizations, or those of the publisher, the editors and the reviewers. Any product that may be evaluated in this article, or claim that may be made by its manufacturer, is not guaranteed or endorsed by the publisher.
